# Transcriptional characterization of relaxin‐3‐positive neurons in aging and Alzheimer’s disease

**DOI:** 10.1002/alz.095602

**Published:** 2025-01-09

**Authors:** Camila de Avila, Jennifer Nolz, Divyanshi Khatri, Zerrin Uzum, Anthony J Intorcia, Geidy E Serrano, Thomas G. Beach, Andrew L Gundlach, Diego Mastroeni

**Affiliations:** ^1^ Arizona State University, Tempe, AZ USA; ^2^ Banner Sun Health Research Institute, Sun City, AZ USA; ^3^ Civin Laboratory for Neuropathology, Banner Sun Health Research Institute, 10515 W Santa Fe Drive, Sun City, AZ 85351, Sun City, AZ USA; ^4^ Florey Institute of Neuroscience and Mental Health, Melboune, VIC Australia

## Abstract

**Background:**

Alzheimer’s disease (AD) is the most prevalent neurodegenerative disorder worldwide and is characterized by clinical symptoms that include deficits in memory and cognition. There is an urgent need to better identify the neural networks that govern cognitive processes in humans and how they are impacted by AD pathology. The brainstem is a critical region that ‘connects’ the forebrain and the spinal cord and contains various nuclei involved in autonomic and complex functions (e.g., locus coeruleus) that are affected during the early stages of AD. In this regard, the brainstem contains the GABAergic nucleus incertus (NI), which has a demonstrated key role in rodents' contextual memory formation by directly inhibiting the hippocampus. Therefore, we mapped the human NI using the neuropeptide relaxin‐3 (RLN3), a neurochemical marker primarily expressed in GABAergic neurons of the NI. In these novel studies, we investigated the transcriptomics of the RLN3‐positive neurons in AD and controls.

**Method:**

Using fresh‐frozen postmortem human tissue provided by the *Banner Brain and Body Donation Program*, Sun City, Arizona, USA, we isolated RLN3‐positive neurons from the NI of non‐demented controls (N = 6), and subsequently from AD (N = 6) age/sex‐matched subjects. After immunostaining, we will use laser‐capture microdissection to isolate RLN3‐positive neurons, followed by RNA sequencing and computational analysis.

**Result:**

Similarly to rodents, we found that RLN3 positive neurons express relaxin/insulin‐like family peptide receptor 3 (*RXFP3*), GABA‐related, cholecystokinin, and corticotropin‐releasing factor type 1 receptor mRNA. In addition, *RLN3* and phosphorylated tau markers, including the microtubule‐associated protein tau, mRNAs were upregulated while *RXFP3* mRNA was downregulated in AD subjects compared to controls. We used RNAscope in situ hybridization as a validation method.

**Conclusion:**

These results suggest that the NI of humans and lower vertebrates share similar neurochemistry. In addition, the NI‐RLN3/RXFP3 system was dysregulated in AD, suggesting RLN3 as a potential biomarker for AD. Further studies investigating RLN3/RXFP3 mRNA changes in pre‐ and early AD are required to understand the time course of its dysregulation.

